# Ghrelin Is Produced in Taste Cells and Ghrelin Receptor Null Mice Show Reduced Taste Responsivity to Salty (NaCl) and Sour (Citric Acid) Tastants

**DOI:** 10.1371/journal.pone.0012729

**Published:** 2010-09-14

**Authors:** Yu-Kyong Shin, Bronwen Martin, Wook Kim, Caitlin M. White, Sunggoan Ji, Yuxiang Sun, Roy G. Smith, Jean Sévigny, Matthias H. Tschöp, Stuart Maudsley, Josephine M. Egan

**Affiliations:** 1 National Institute on Aging/National Institutes of Health, Baltimore, Maryland, United States of America; 2 Huffington Center on Aging, Baylor College of Medicine, Houston, Texas, United States of America; 3 Department of Metabolism and Aging, The Scripps Research Institute, Scripps Florida, Jupiter, Florida, United States of America; 4 Centre de Recherche en Rhumatologie et Immunologie, Centre Hospitalier Universitaire de Québec, Université Laval, Québec City, Québec, Canada; 5 Division of Endocrinology, Departments of Medicine and Psychiatry, Metabolic Diseases Institute, University of Cincinnati College of Medicine, Cincinnati, Ohio, United States of America; Pennsylvania State University, United States of America

## Abstract

**Background:**

The gustatory system plays a critical role in determining food preferences, food intake and energy balance. The exact mechanisms that fine tune taste sensitivity are currently poorly defined, but it is clear that numerous factors such as efferent input and specific signal transduction cascades are involved.

**Methodology/Principal Findings:**

Using immunohistochemical analyses, we show that ghrelin, a hormone classically considered to be an appetite-regulating hormone, is present within the taste buds of the tongue. Prepro-ghrelin, prohormone convertase 1/3 (PC 1/3), ghrelin, its cognate receptor (GHSR), and ghrelin-O-acyltransferase (GOAT , the enzyme that activates ghrelin) are expressed in Type I, II, III and IV taste cells of mouse taste buds. In addition, ghrelin and GHSR co-localize in the same taste cells, suggesting that ghrelin works in an autocrine manner in taste cells. To determine a role for ghrelin in modifying taste perception, we performed taste behavioral tests using GHSR null mice. GHSR null mice exhibited significantly reduced taste responsivity to sour (citric acid) and salty (sodium chloride) tastants.

**Conclusions/Significance:**

These findings suggest that ghrelin plays a local modulatory role in determining taste bud signaling and function and could be a novel mechanism for the modulation of salty and sour taste responsivity.

## Introduction

The mouth is the first section of the alimentary canal that receives and experiences food. It begins digestion by mechanically breaking food into smaller pieces and mixing them with saliva to facilitate swallowing. Additionally, the mouth is also part of the gustatory system and taste cells (TCs) in taste buds of the tongue engender distinct taste perception qualities. On the basis of these perceptions, further food intake is then considered to be, or not to be, desirable. There are five basic taste modalities: bitter, sweet, umami [the savory taste of monosodium glutamate (MSG)], salty, and sour. Sweet, umami and salt modalities allow recognition of energy-containing nutrients and maintenance of electrolyte balance, while sour and bitter taste modalities are thought to act as brakes or warnings against further ingestion of rancid or noxious foods.

Processing of taste begins with molecular events at the surface membranes of modified epithelial-derived TCs, which are organized in taste buds within circumvallate (CV), foliate and fungiform papillae [1]–[3]. Mammals have four types of TCs (Types I, II, III, and IV) within their taste buds and these cell types exhibit different molecular phenotypes and functional roles. Type I cells are glial-like cells that maintain taste bud structure [4]. Type II TCs transduce sweet, bitter, or umami stimuli [5], and utilize a G protein-coupled transduction cascade for signaling [2], [3]. Type III cells synapse directly with afferent nerve fibers from three cranial nerves [6], and most release serotonin (5-hydroxy-tryptamine; 5-HT) upon depolarization [7]. Finally, Type IV cells (sometimes called basal cells) are rapidly dividing progenitor cells that differentiate into the other types of TCs [8]. Knowledge of sour and salty transduction machinery has recently greatly expanded. Polycystic kidney disease 2-like 1 (PKD2L1) and polycystic kidney disease 1-like 3 (PKD1L3), two members of the transient receptor potential channel family, have been identified in a subset of TCs distinct from sweet, umami or bitter cells [2] and PKD2L1-positive cells co-express various Type III cell marker proteins [9]–[12]. Ablation of these cells has been shown to cause a selective loss of behavioral responses to only sour stimuli, such as citric acid (CA), indicating that PKD2L1-expressing Type III cells play a role in transducing sour taste [2]. However, it must be noted that no specific taste behavioral tests were performed in this study. Even more recently, salt sensation was shown to be mediated, in-part, through epithelial sodium channels (ENaC) [13]–[15].

Effective and discrete gustation is vital for determining which foods are suitable to ingest, and for maintaining body weight and energy balance. It is becoming apparent that there is a strong link between peripheral energy balance and ‘flavor perception’. We recently reported that glucagon-like peptide-1 (GLP-1), typically considered as an incretin hormone produced by the enteroendocrine cells of the gut (whose peripheral function is to regulate insulin secretion and gastric emptying), is also produced in TCs [16]. Disruption of GLP-1 signaling in mice causes a significantly decreased sensitivity to sweet tastants, and increased sensitivity to umami and sour tastants. Therefore hormones which were classically considered to be gut and appetite hormones, are also produced by TCs where they may play a modulatory role in fine tuning taste perception [17]–[19].

In our previous study, we found that the enzyme prohormone convertase 1/3 (PC1/3) which cleaves pro-glucagon into GLP-1 is present within TCs. There were however significantly more PC1/3-expressing TCs than GLP-1-expressing TCs [16], which prompted us to investigate which additional PC1/3 substrates might be present within TCs. Ghrelin, a 28-amino-acid peptide, is an orexigenic hormone that was first isolated from X/A cells in the stomach [20] and is a ligand for the growth hormone secretagogue receptor (GHSR) [21]. Ghrelin expression is not limited to the stomach, but is found at many other sites such as the small intestine, brain, pituitary, lung, skeletal muscle, islets of Langerhans, adrenal glands, ovary, and testis [22]. Ghrelin has also been shown to be produced by human salivary glands and is secreted into saliva [23]. Similar to many other peptide hormones, ghrelin is processed from a larger precursor (94-amino-acid) by PC1/3, which appears to be the only enzyme involved in the processing of ghrelin *in vivo* [24], [25]. To activate the GHSR, ghrelin must be acylated with an eight-carbon fatty acid at serine 3 by ghrelin-O-acyltransferase (GOAT) [26], [27]. After ghrelin and GHSR were discovered [20], [21], [28], it quickly became apparent that besides its capacity to release growth hormone, ghrelin also has many other actions linked to feeding behavior, energy homeostasis, reproduction, sleep regulation, corticotrope secretion and regulation of gastro-entero-pancreatic functions [29]–[31]. Despite this plethora of effects however, mice in which ghrelin and GHSR were knocked out demonstrate normal growth, energy expenditure, and food intake under normal chow conditions [32]–[34], suggesting that ghrelin plays primarily a facilitatory role in several complex endocrine axes. In this study, we demonstrate that ghrelin is produced within the TCs of the tongue in PC 1/3-expressing cells, that GHSR is expressed on TCs, and using GHSR null mice, we show that ghrelin plays a role in modifying specific taste qualities.

## Results

### Ghrelin and the prepro-ghrelin cleaving enzyme, PC 1/3, are present in TCs of the tongue

**Figure 1:**
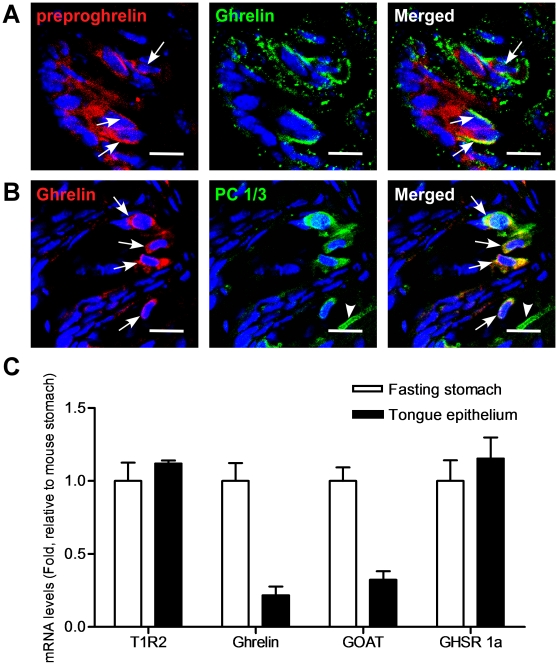
Expression of a prepro-ghrelin, PC1/3, and ghrelin in circumvallate papillae (CV) taste cells of mice. (A) preproghrelin and ghrelin are co-localized in a subset of taste cells. Arrows, cells expressing both proteins. (B) ghrelin and PC1/3 are co-expressed in a subset of PC 1/3-positive cells. Arrowhead, cells expressing only PC 1/3; arrows, cells expressing both. Scale bars, 20 µm. Blue is TO-PRO-3 nuclear stain. (C) Quantitative real-time PCR of cRNA from tongue and stomach. Experiments were carried out in triplicate and replicated at least twice. Values are expressed as means ± S.E.M.

**Figure 2:**
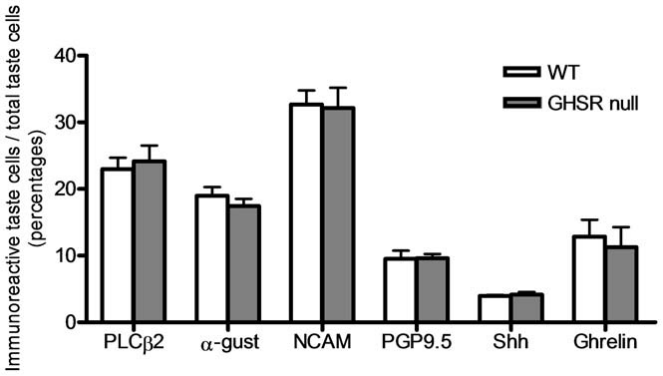
Comparison of cell types in circumvallate papillae of wild type and GHSR null mice. The total number of cells in the section was determined by counting the number of TO-PRO-3 stained nuclei present in each taste bud. Percentage of immunoreactive taste cells was calculated by dividing the number of immunoreactive taste cells by the total number of the taste cells in each taste bud. Values are expressed as means ± S.E.M.

**Figure 3:**
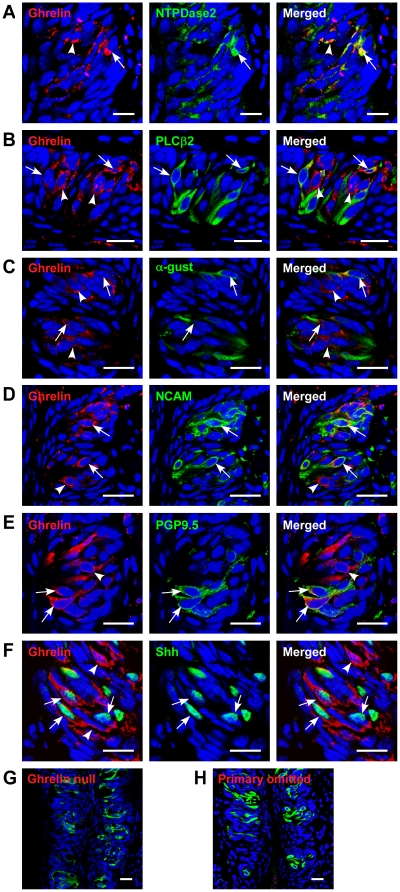
Co-expression of ghrelin and taste cell markers in circumvallate papillae (CV) of mice. (A) ghrelin and NTPDase2 are co-localized in a subset of NTPDase2-positive cells. Arrowhead, cell expressing only ghrelin; arrow, cell expressing both. (B) ghrelin and PLCβ2 are co-localized in a subset of PLCβ2-positive cells. Arrowhead, cell expressing only ghrelin; arrow, cells expressing both. (C) ghrelin and α-gustducin are co-localized in a subset of α-gustducin-positive cells. Arrowhead, cell expressing only ghrelin; arrows, cell expressing both. (D) ghrelin and NCAM are co-localized in a subset of NCAM-positive cells. Arrowhead, cell expressing only ghrelin; arrow, cell expressing both. (E) ghrelin and PGP9.5 are co-localized in a subset of PGP9.5-positive cells. Arrowhead, cell expressing only ghrelin; arrow, cell expressing both. (F) ghrelin and Shh are co-localized in a subset of Shh-positive cells. Arrowhead, cell expressing only ghrelin; arrows, cells expressing both. (G) ghrelin and PLCβ2 are stained in ghrelin null mice as a negative control. (H) ghrelin and PLCβ2 are co-stained after the ghrelin antibody was omitted. Scale bars, 20 µm. Blue is TO-PRO-3 nuclear stain.

The vast majority of ghrelin that is assayed in plasma originates from X/A cells in the stomach [20]. We have now found that ghrelin and its precursor molecule, prepro-ghrelin, are present in 13±3% of all TCs in mouse CV (Figure 1A and 2). Additionally these cells, as expected, were also immunopositive for the prepro-ghrelin cleaving enzyme, prohormone convertase 1/3 (PC1/3) (Figure 1B). To corroborate our immuno-fluorescence data, we used quantitative real-time PCR to demonstrate that ghrelin, GOAT and GHSR mRNA, were expressed in both tongue and stomach epithelium, as expected (Figure 1C, See Table S1 for primer sequences). This indicates that the ghrelin precursor prepro-ghrelin, ghrelin, the ghrelin-cleaving enzyme PC1/3, and GOAT are all expressed in TCs. We further investigated the qualitative nature of the ghrelin-positive TCs with classical taste cell marker identification. Details of the antibodies used and the specific TC markers are given in Table S2 [8], [25], [35]–[43]. Briefly, we used Nucleoside triphosphate diphosphohydrolase-2 (NTPDase2) antibody as a Type I TC marker [39], α-gustducin and Phospholipase C-type β2 (PLCβ2) antibodies as Type II TC markers [40], [41]. Neural Cell Adhesion Molecule (NCAM) was employed as a Type III marker [42]. Protein gene product 9.5 (PGP9.5) was used as both a Type II and III cell marker as well as an indicator of nerve fibers [44]. ENaC was used as a marker of salt taste-expressing cells [13], [14] and Sonic hedgehog (Shh) as a Type IV cell marker [8]. In circumvallate papillae, we found that approximately one-quarter of all ghrelin-positive cells were Type II (27±3%), one-third were Type III (33±4%) and the remaining were Type I (29±6%) and IV (10±4%) cells (Figure 3 and Table 1). Similar results were obtained for foliate papillae (Figure S1 and Table S3).

**Table 1:** Co-localization of immunocytochemical markers in mouse circumvallate papillae taste cells.

Co-localized Marker	Co-localized taste cells/total ghrelin-positive taste cells (Percentages, Mean ± S.E.M.)		
	WT mice	GHSR null mice	P value
PLCβ2	27±3	29±5	0.165
α-gustducin	25±8	27±5	0.316
NCAM	33±4	42±9	0.103
PGP9.5	22±4	21±7	0.416
Shh	10±4	14±1	0.207
Rest of TCs	29±6	16±4	0.068

### GOAT and GHSR are expressed in the TCs of WT mice

**Figure 4:**
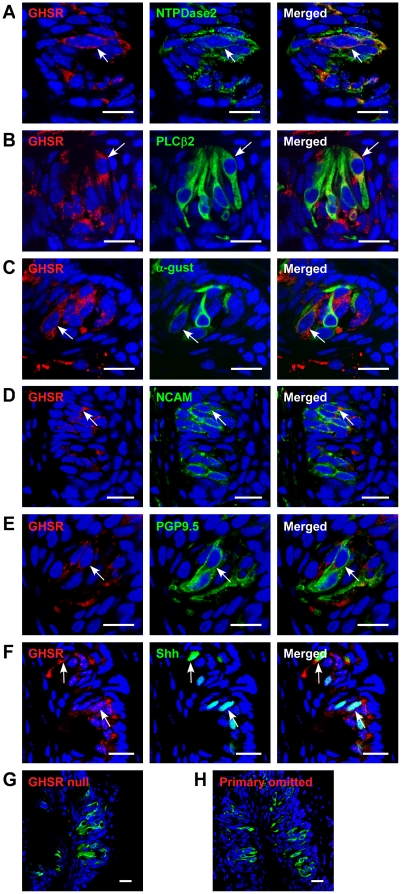
Co-expression of GHSR and taste cell markers in circumvallate papillae (CV) of mice. (A) GHSR and NTPDase2 are co-localized in a subset of NTPDase2-positive cells (arrow). (B) GHSR and PLCβ2 are co-localized in a subset of PLCβ2-positive cells (arrow). (C) GHSR and α-gustducin are co-localized in a subset of α-gustducin-positive cells. Arrow, cells expressing both. (D) GHSR and NCAM are co-localized in a subset of NCAM-positive cells. Arrow, cell expressing both. (E) GHSR and PGP9.5 are co-localized in a subset of PGP9.5-positive cells (arrow). (F) GHSR and Shh are co-localized in a subset of Shh-positive cells (arrows). (G) GHSR and PLCβ2 are stained in ghrelin null mice as a negative control. (H) GHSR and PLCβ2 are co-stained after the ghrelin antibody was omitted. Scale bars, 20 µm. Blue is TO-PRO-3 nuclear stain.

**Figure 5:**
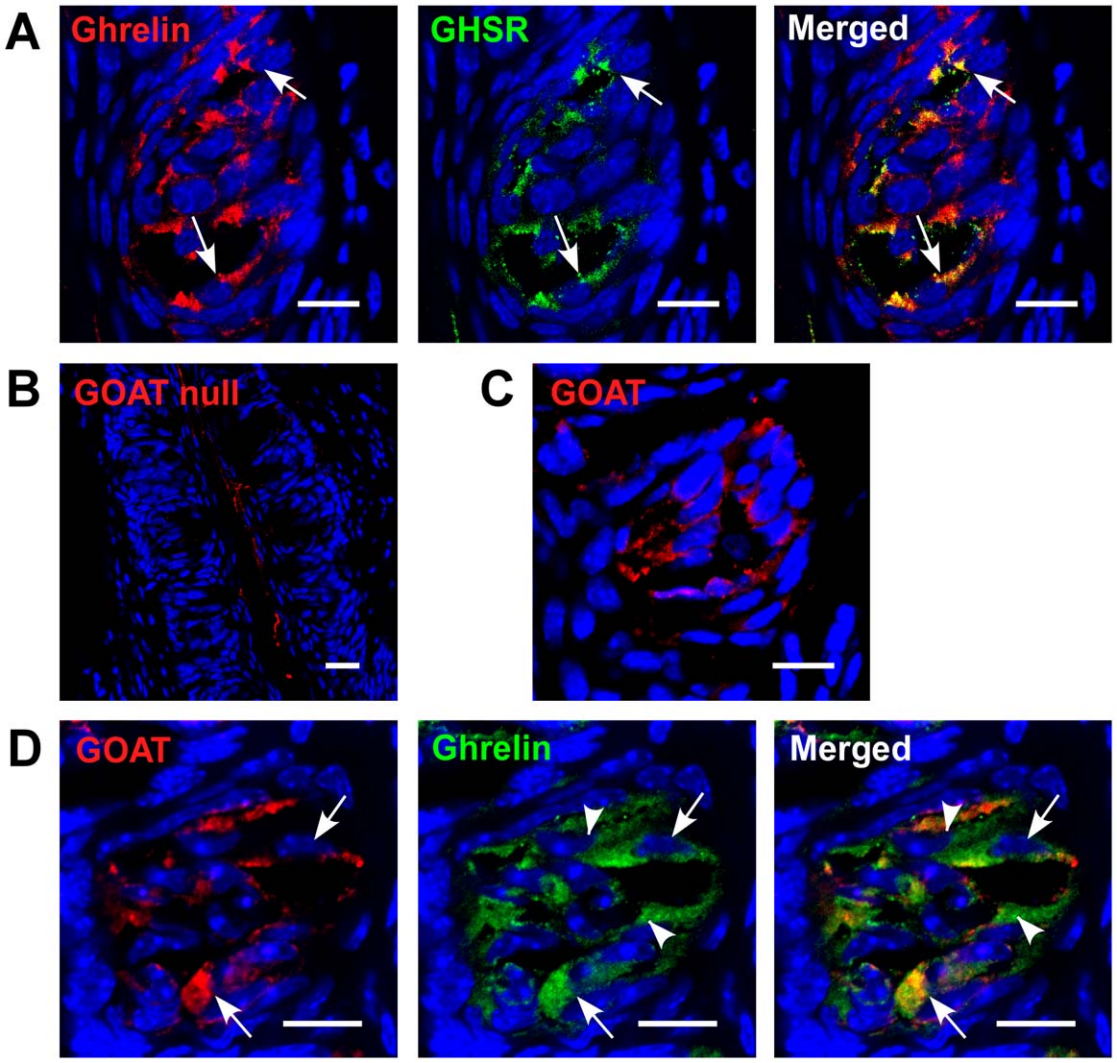
Co-expression of ghrelin/GHSR and ghrelin/GOAT in circumvallate papillae (CV) of mice. (A) Ghrelin and GHSR are co-localized in a subset of ghrelin-positive cells (arrows). (B) GOAT is stained in GOAT null mice as a negative control. (C) GOAT is expressed in TCs. (D) GOAT and ghrelin are co-localized in a subset of ghrelin-positive cells (arrows). Scale bars, 20 µm. Blue is TO-PRO-3 nuclear stain.

We next investigated whether the GHSR was expressed in TCs. We demonstrated that the GHSR was expressed on all four types of TCs in CV and foliate papillae (Figure 4, Figure S2). The majority of cells that contained the GHSR also expressed ghrelin (Figure 5A–C).

To be functional, ghrelin needs to be acylated by GOAT. We found that GOAT and ghrelin are also co-localized in many cells, however we did also identify ghrelin immunopositive cells in which no GOAT was expressed (Figure 5D). We found that 4% of total taste cells were GOAT immunopositive, 13% of total taste cells were ghrelin immunopositive, and both GOAT and ghrelin were co-expressed in 4% of taste cells. The fact that ghrelin and its cognate receptor are co-expressed in the same TC suggests that ghrelin signaling is locally active in an autocrine manner in taste buds and suggests that ghrelin signaling in the taste bud may functionally affect taste perception.

### The GHSR in the TCs regulates multiple taste qualities

Before we investigated taste perception in the wild-type (WT) and GHSR null mice, we first determined whether the GHSR null mice had altered taste bud morphology, size or TC numbers. Taste buds were in the same locations in CV and foliate papillae (Figures S1 and S3), cellular appearances were similar, and mean taste bud area (WT: 1,336±41 mm^2^ vs. null: 1,342±46 mm^2^, p = 0.926) and taste cell numbers per taste bud (WT: 47±3 vs. null: 45±6, p = 0.749) were not altered in the papillae of GHSR null mice. The number of taste marker immunopositive cells and their cellular distribution showed a similar expression pattern in CV and foliate papillae in both the WT and GHSR null mice (Figures 2, S1, S3 and Table S4). Finally, we examined the distribution of ghrelin-positive cells with the four TC makers in GHSR null mice (11±3% of TCs in taste buds of null mice were positive for ghrelin immunostaining) and we found no statistical significance between WT and GHSR null mice in either CV (Figures 3, S3 and Table 1) or foliate papillae (Figure S1 and Table S3).

**Figure 6:**
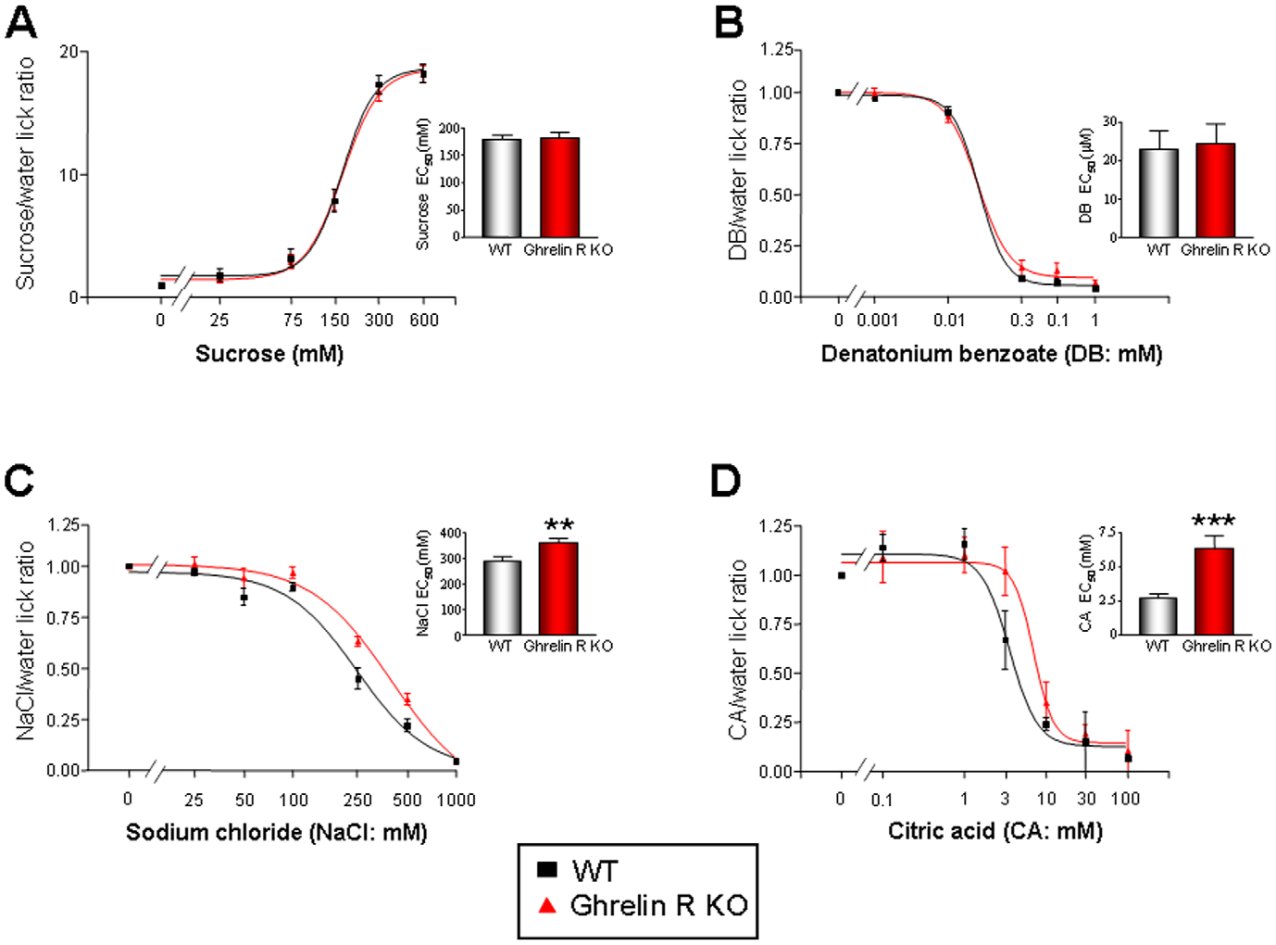
Altered salt and sour taste responses of WT and GHSR null mice in brief access taste tests. Taste responses, expressed as tastant/water lick ratios and as a function of stimulus concentration, of GHSR null, (red) and WT (black) to (A) sucrose, (B) DB, (C) NaCl and (D) CA. Points are expressed as means ± S.E.M. Curves were fit as described in Methods. **p<0.01; ***p<0.001.

We tested the ability of WT and GHSR null mice to detect four prototypic tastants, *i.e.* sweet (sucrose), sour (CA), salty (sodium chloride – NaCl), and bitter (denatonium benzoate – DB). For this we used a computer-controlled gustometer, involving a brief-access procedure that minimizes post-ingestive effects [45]. There were no significant differences between the responses of the GHSR null mice and age-matched WT controls (n = 8 for each genotype) for sucrose or DB (Figures 6A, 6B, S4A, and S4B - log representation). Interestingly however, the GHSR null mice displayed a significantly reduced sensitivity to NaCl and CA (NaCl p<0.01; CA p<0.001) when compared to the WT mice (Figures 6C, 6D, S4C, and S4D - log representation). While the significant differences in the mean EC_50_s calculated for NaCl and CA were small, it should be noted that the animals demonstrated a relatively narrow tastant sensitivity range and therefore large logarithmic differences in perception between WT and GHSR null mice may be difficult to achieve as other peptides are also likely to also control these modalities [46]. Taken together, these results suggest that ghrelin signaling plays a functional role in modulating taste responsivity linked to sour and salty taste modalities.

**Figure 7:**
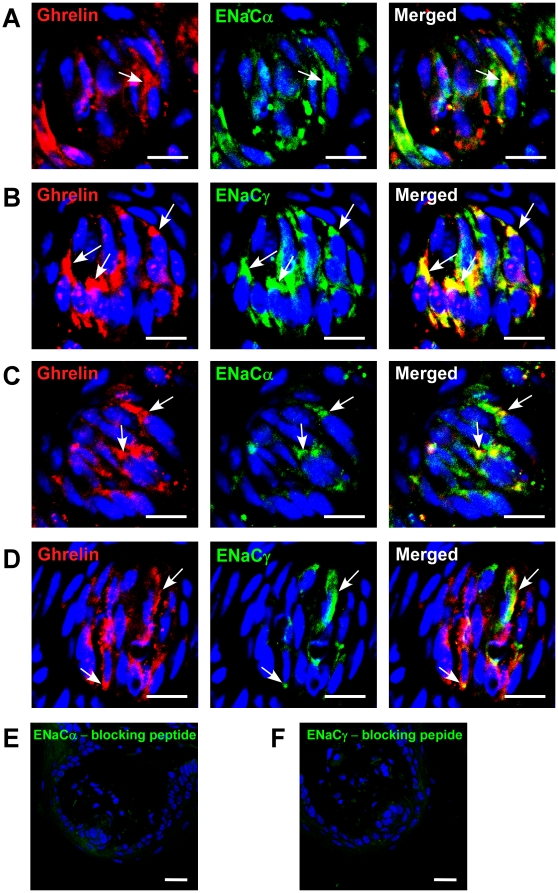
Co-expression of ghrelin and ENaC subunits in fungiform papillae of WT (A,B) and GHSR null (C,D) mice. (A) ghrelin and ENaCα are co-localized in a subset of ghrelin-positive cells in WT mice. Arrow, cell expressing both. (B) ghrelin and ENaCγ are co-localized in a subset of ghrelin-positive cells in WT mice. Arrows, cells expressing both. (C) ghrelin and ENaCα are co-localized in a subset of ghrelin-positive cells in GHSR null mice. Arrows, cells expressing both. (D) ghrelin and ENaCγ are co-localized in a subset of ghrelin-positive cells in GHSR null mice. Arrows, cells expressing both. (E) ENaCα staining after treatment with blocking peptide. (F) ENaCγ staining after treatment with blocking peptide. Scale bars, 20 µm. Blue is TO-PRO-3 nuclear stain.

We found that ghrelin and GHSR are expressed in the fungiform papillae (Figures S5A and S5B). Salt sensitivity is thought to be modulated by fungiform papillae [14]. Thus, we first investigated whether the reduced NaCl sensitivity that we observed in our GHSR null mice could be due to a reduction in the overall number of papillae. This was not the case (WT: 104±5 vs. GHSR null: 100±5, p = 0.675), so we next investigated whether there were any alterations in expression of ENaC subunits in the taste buds of fungiform papillae. We found that ENaC alpha and gamma subunits were both co-expressed with ghrelin in TCs in both the WT and GHSR null mice (Figure 7). Interestingly, we found a marked reduction in the number of TCs expressing ENaC subunits (∼50%) in the GHSR null mice. Finally, in order to ascertain whether our results from rodents could also be applicable to primates, we examined a CV from a rhesus monkey and we also found evidence of expression of ghrelin and its receptor (Figures S5C and S5D).

## Discussion

We have shown that prepro-ghrelin, PC 1/3, GOAT, ghrelin, and GHSR are present within TCs of the mouse taste bud and that ghrelin may play a functional role in modulating CA (sour) and NaCl (salty) tastes. To be biologically active, ghrelin needs to be acylated by GOAT. GOAT is a member of the MBOAT (membrane bound O-acyl transferase) family that acylates ghrelin at serine-3, in a process that involves four amino acids at the N-terminal [27], [47], [48]. Additionally, GOAT can also use other fatty acid substrates to acylate ghrelin, such as n-hexanoyl-CoA [48]. *In vivo* studies have demonstrated that GOAT gene disruption in mouse models can completely abolish ghrelin acylation [47]. We found that GOAT and ghrelin are co-localized in many, but not all, taste cells. Therefore a subset of ghrelin immunopositive cells was found to contain no discernable GOAT expression. We found that 4% of total taste cells were GOAT immunopositive, 13% of total taste cells were ghrelin immunopositive, and both GOAT and ghrelin were co-expressed in 4% of taste cells. This observation could explain why the GHSR null mice only showed alterations in taste sensitivity to salty and sour tastants, even though ghrelin and GHSR were expressed in all 4 cell types.

The exact mechanisms that fine tune salt taste transduction are presently not well understood. It is known that salt taste transduction in taste cells is composed of at least two different systems that are amiloride-sensitive (AS) and amiloride-insensitive (AI). These two systems are distinguished by their sensitivity to the epithelial Na^+^ channel blocker, amiloride [49]–[51]. The specific cell type populations that modulate NaCl sensitivity are presently unclear. A recent study showed that AS Na^+^ channels were expressed in type I taste cells, which lack voltage-gated inward currents [52]. ENaC is composed of three essential subunits, α-, β-, and γ-ENaC subunits, and all three subunits are required for activity of this channel [53], [54]. We found co-expression of ghrelin with the ENaC α- and γ subunits and we also found reduced numbers of cells containing ENaC subunits in GHSR null mice. Taste cells expressing functional AS channels have also been shown to possess large voltage-gated sodium currents that underlie the generation of action potentials [55]. A third study has demonstrated that AS cells can generate action potentials and express ENaC subunits, suggesting that AS cells may be taste cells distinct from Type I cells without action potentials [56]. However, this study did not detect expression of α-gustducin, a marker for type II cells, or synaptosomal-associated protein 25 (SNAP25), a marker for type III cells, in the AS cells. Thus, it is possible that these AS cells could be type II or type III cells which do not express gustducin or SNAP25 [56]. This suggests that salt taste transduction could be modulated by multiple taste cell types, including types I, II and III. In addition to the epithelial Na^+^ channel, there are also likely to be additional NaCl transduction mediators, including transient receptor potential cation channel, subfamily V, member 1 (TRPV1) [57] and nitric oxide [58]. It is evident that further research is needed to elucidate the exact mechanisms that fine tune salt taste transduction and which additional hormones potentially play a role in modulating salt taste sensitivity. It is intriguing to note that with regards to our observed taste phenotype in the GHSR null mice, *i.e.* disrupted salt and sour responsivity, it has been demonstrated that a subset (45%) of salt-sensing taste cells (AI) cells have been identified that share multiple phenotypic characteristics (SNAP25, PKD2L1) of sour-sensing cells [56]. The presence of this sub-population of TCs, if affected by ghrelin signaling, could account for the complex and unexpected taste phenotype we have observed. This possibility is likely to be an important future field of research with regards to neuropeptide signaling in the tongue.

The ability of ghrelin receptor ablation to affect the response to a sour stimulus could potentially be due to a role of ghrelin in controlling cell to cell signaling between TCs and the Type III (‘presynaptic’) cells, which possess the putative sour receptor (PKD2L1). Recent data has shown that while presynaptic cells do not possess tastant-sensitive G protein-coupled receptors, they can still respond to tastant stimulation [5]. Communication between TCs and presynaptic cells is thought to involve adenosine-5′-triphosphate (ATP) and serotonin secretion from the TCs [41]. Ghrelin binds to the GHSR to promote foraging and feeding behaviors, mainly via the hypothalamic arcuate nucleus (ARC). GHSR is also expressed in the midbrain dopaminergic neurons of the ventral tegmental area (VTA), suggesting that ghrelin may play a role in regulating the mesolimbic system. It has been shown that ghrelin can increase serotonin levels in the shell subdivision of the nucleus accumbens [59]. Thus, it could be possible that in the tongue ghrelin could be acting in an analogous manner as in other parts of the central nervous system, *i.e*. in a concerted manner with serotonin. Genetic disruption therefore of ghrelin signaling could affect efficient TC-presynaptic cell communication, resulting in the reduction in sour sensitivity we observed in GHSR null mice.

While we cannot conclusively presume that the effect of ghrelin in the TCs is a direct, paracrine effect, several lines of evidence suggest that this may be the case. Firstly, TCs express both ghrelin and its receptor. Secondly, in the GHSR null mice we demonstrated a hyposensitivity to only CA (sour) and NaCl (salty) tastes, indicating that—despite ghrelin and GHSR being expressed in all four TC types—there was not a widespread effect on all taste qualities. This was likely due to the fact that not all ghrelin immunopositive cells co-expressed GOAT. Thirdly, the GHSR null mice responded similarly to the WT mice for the sweet and bitter tastants, suggesting that the GHSR null mice had no difficulty learning or completing the taste-testing task. Future studies are needed that may definitely demonstrate that ghrelin (and other additional hormones expressed in TCs) is released from TCs. Current limitations in available technology to measure active, functional hormone release from TCs in response to tastants, need to be overcome to facilitate these measurements, as current methods all have significant logistical flaws. Direct *in vivo* measurements will potentially be contaminated by ghrelin present in saliva from salivary glands and circulating in high concentrations in the blood stream. Electron microscopy analyses of ghrelin in secretory vesicles, while revealing, would not provide conclusive evidence that ghrelin is actually secreted from the TCs during taste perception. Considerable technological advancement may be necessary to overcome these present challenges.

In light of our previous findings, *i.e.* that the gut hormone GLP-1 is present within TCs and strongly modulates sweet and umami taste sensitivity [16], [60], it is now becoming apparent that many of the classically considered appetite and gut hormones [57], [61] are present within TCs of the tongue and play important functional roles in TC physiology. Gaining a greater understanding of which endocrine factors are present within TCs, their putative roles in taste bud signaling and overall taste perception will shed some much needed light on how taste sensitivity is fine tuned and how taste perception is linked to peripheral energy balance. Future work will hopefully further elucidate which additional hormones are present within TCs and uncover the specific functional roles that each hormone exhibits in modulating taste transduction and taste bud physiology.

## Materials and Methods

### Animals and tissue processing

All animal testing procedures were approved by the Animal Care and Use Committee of the National Institute on Aging (NIA; Protocol number: 156-LCI-JME). Male ghrelin receptor knockout mice on a BL6/C57 background and their WT BL6/C57 counterparts were employed for our studies (n = 8 for all behavioral studies) [32]. All animals used in this experiment were littermates, and all animals were on the same genetic background (*i.e*. BL6C57).

For antibody validation, ghrelin knockout mice [62] and GOAT knockout mice were employed for our studies [63]. After taste testing was completed with GHSR null mice, animals were anesthetized using isoflurane and the tongue, pancreas and stomach were collected from each animal. Excised tongues were fixed in 4% paraformaldehyde (Sigma, St. Louis, MO) for 1 hour and then cryoprotected with 20% sucrose in 0.1 M phosphate buffer overnight at 4°C. Serial sections (8–10 µm thickness) were cut from the tissues containing circumvallate, foliate and fungiform papillae, using a cryostat (HM 500 M, MICRON, Laborgerate GmbH, Germany). A portion of freshly-dissected adult rhesus monkey tongue containing a CV papilla was obtained from the NIA monkey colony, as the monkey was undergoing euthanasia and autopsy (Protocol number: 379-LEG-2010).

### Isolation of tongue epithelium

The dorsal epithelium of rodent tongue, containing both anterior and posterior taste fields, was isolated using a previous protocol [64]. The peeled epithelium was stored at −70°C for RNA isolation. In addition the stomach was also stored at −70°C for similar procedures.

### RNA isolation and real-time PCR of taste buds and stomach

Total RNA was extracted using Trizol reagent (Invitrogen) from lingual epithelium and stomach according to the manufacturer's instructions. After reverse transcription, the resulting materials were used for PCR amplification using gene-specific primer pairs (Table S1) and SYBR Green PCR master mix (Applied Biosystems, Foster City, CA). For real-time PCR, amplification conditions were 50°C (2 min), 95°C (10 min), and then 40 cycles at 95°C (15 sec) and 60°C (1 min) [65]. The data were normalized to glyceraldehyde 3-phosphate dehydrogenase (GAPDH) mRNA. All real-time PCR analyses are represented as the means ± standard errors of the means (S.E.M.) from at least three independent experiments, each performed in triplicate.

### Immunohistochemistry

After antigen retrieval with 1x citrate buffer (Biogenex, San Ramon, CA) at 98°C for 20 min, immunofluorescence analyses were performed as described previously [39], [66]. Cryostat sections were blocked in 5% bovine serum albumin (BSA; Sigma) and 0.1% Tween-20 in 1X Tris-buffered saline (TBS) (pH 7.4) for one hour at room temperature, followed by incubation in a specific primary antibody in 1% BSA and 0.1% Tween-20 in TBS (pH 7.4) overnight at 4°C. Sources and dilutions of the applied primary antibodies are listed in Table S2.

We used ghrelin, GHSR and GOAT null mice to validate our antibodies. No specific immunocytochemical signal was detected in the ghrelin, GHSR or GOAT null mice with our antibodies raised against these respective proteins, suggesting that the antibodies were indeed specific. Additionally, we also used ghrelin blocking peptide as an additional control to test the specificity of the anti-ghrelin antibody. (Figure 3G, 4G and 5B). After washing, sections were incubated for 1 hour in fluorescent secondary antibodies (FITC, Rhodamine Red-X, (1∶1000 dilution; Jackson ImmunoResearch, West Grove, PA)) along with TO-PRO-3 (1∶7000 dilution; Molecular Probes, Carlsbad, CA), in some cases, for nuclear staining. No fluorescent staining was observed in any sections when the primary antibodies were omitted (Figure 3H and 4H).

### Quantification of immunoreactive taste cells

In order to obtain a systematic sample without bias throughout the papillae, each papilla was exhaustively sectioned and every tenth section was saved onto a slide. A taste bud is approximately 80–100 µm in length and so sampling every tenth section will ensure that no two sections will be from the same taste bud. Confocal images were collected using an LSM-410 and LSM-710 confocal microscope (Carl Zeiss MicroImaging, Thornwood, NY) in single planes. Approximately 100–120 taste buds per group were analyzed, as described previously [40]. Cells were scored as immunoreactive only if a nuclear profile was present within the cell. The total number of cells in the section was determined by counting the number of TO-PRO-3 stained nuclei present in each taste bud. Finally, the percentage of immunoreactive taste cells was calculated by dividing the number of immunoreactive taste cells by the total number of the taste cells in each taste bud. The data were collected in a blinded fashion. One of the investigators scored samples that had ID numbers on the animals. After quantification was complete, the animal IDs were matched to the phenotype.

### Quantification of taste bud size

To calculate taste bud size, the perimeters of the taste bud from every tenth section were outlined and the corresponding area was computed by Zeiss LSM image browser [16]. At the same time, 20 taste buds were randomly selected in different regions of the tongue per animal to count cells in a single taste bud, and 1 nucleus corresponded to 1 cell on the section.

### Taste behavioral tests and data analysis

The taste behavioral testing was performed as previously described [16]. All taste testing took place during daylight hours. GHSR null (n = 8) and WT (n = 8) mice were habituated to the laboratory environment for 30 minutes each day prior to the initiation of taste testing. All tastants were prepared with purified water from the NIA animal facility and reagent grade chemicals and were presented to the animals at room temperature. Test stimuli consisted of various concentrations of sucrose (25, 75, 150, 300, and 600 mM; Fisher Scientific, Atlanta, GA, USA), NaCl (25, 50, 100, 200, 250, 500, 1000 mM; Sigma-Aldrich, St. Louis, MO, USA), DB (0.001, 0.01, 0.1, 0.3, and 1 mM; Sigma-Aldrich, St. Louis, MO, USA), and CA (0.01, 0.1, 1, 3, 10, 30, and 100 mM; Fisher Scientific). Brief-access taste testing took place in a Davis MS-160 gustometer (DiLog Instruments, Tallahassee, FL, USA), as previously described [39], [67]–[70]. Brief-access procedures minimize post-ingestive effects that may confound other assays such as intake tests [39]. Mice accessed the taste stimuli (presented as a concentration range) or water in sipper bottles through a small opening in the mouse chamber. Before taste testing was initiated, mice were trained to lick a stationary tube of water in the gustometer after being placed on a 23.5 hour restricted water-access schedule. Unconditioned licking responses were recorded for later analyses in 25 minute brief-access test sessions, during which mice could initiate as many trials as possible in this period. Stimulus presentation order was randomized within blocks. The duration of each trial (5 seconds) was regulated by a computer-controlled shutter that allowed access to the sipper tube. There was a 7.5 second inter-presentation interval, during which time a stepper motor moved one of up to seven tubes (containing water or a specific concentration of tastant) in front of the shuttered opening. Two different testing protocols were used: one for normally preferred stimuli (sucrose) and one for normally avoided stimuli (NaCl, DB, and CA). For sucrose, animals received 5 days of testing using the five stimulus concentrations and purified animal facility water. Prior to each day of sucrose testing, animals were placed on a 23.5 hour restricted food and water-access schedule (1 gram of food and 2 ml of water) in order to maintain motivation to drink, and thus increasing the number of stimulus presentations taken during testing [69], [70]. In a similar manner for the other tastants, NaCl, CA and DB, animals received 5 days of testing with the five stimulus concentrations and with purified animal facility water. Similarly to the testing performed with sucrose, the mice were water-deprived during NaCl, DB, and CA testing in order to increase the number of stimulus presentations taken. Additionally, a water rinse presentation (1 s) was interposed between the test trials for NaCl, DB, and CA to help control for any potential tastant carry-over effects.

### Data analysis and statistical methods for behavioral testing

The average number of licks per trial for each stimulus concentration was divided by the average number of water licks per trial, yielding a tastant/water lick ratio. This ratio controls for individual differences in motivational state [68]. The ratios were analyzed with standard ANOVA and t-test. When a genotype × concentration interaction was significant, one-way ANOVA was conducted within each genotype to test for simple effects. The conventional p≤0.05 was applied as the statistical rejection criterion. Tastant concentration-lick ratio response curves were fitted to the mean data for each group using a classical four parameter logistic sigmoidal dose-response equation using the non-linear regression suite of GraphPad Prism (v3.0). For this relationship y = (max-min)/(1+10
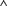
(log_10_EC_50_-x) * Hill slope)), where x is the log_10_ of the concentration and y is the response starting at the minimum (min) and ending at the maximum (max). The EC_50_ value is the concentration at which a 50% of maximal response is attained. Using this relationship effect, individual animal EC_50_ values were obtained each day and compared between WT animals and GHSR null animals. The EC_50_ values were calculated for each animal from the entire curve. Data with goodness of sigmoidal curve fits (r^2^) of less than 0.95 were rejected. Statistically significant differences between EC_50_s obtained for WT or GHSR null mice were assessed using a non-paired student's t-test (GraphPad Prism).

## Supporting Information

Figure S1 Co-expression of ghrelin in mouse foliate papillae of WT (A–E) and GHSR null (F–J) mice. (A, F) ghrelin is co-expressed with PLCβ2. (B, G) ghrelin is co-expressed with α-gustducin. (C, H) ghrelin is co-expressed with NCAM. (D, I) ghrelin is co-expressed with PGP9.5. (E, J) ghrelin is co-expressed with Shh. Scale bars, 20 µm. Blue is TO-PRO-3 nuclear stain.
(3.68 MB TIF)

Figure S2 Co-expression of GHSR in foliate papillae of wild type (A–E) and islets of Langerhans (F–H) of wild type and GHSR null mice. (A) GHSR is co-expressed with PLCβ2. (B) GHSR is co-expressed with α-gustducin. (C) GHSR is co-expressed with NCAM. (D) GHSR is co-expressed with PGP9.5. (E) GHSR is co-expressed with Shh. (F) In islets, GHSR is co-expressed with insulin-containing cells (yellow); therefore in islets GHSR is expressed on cells. (G) GHSR (red) is not expressed in glucagon-containing (green) cells (no yellow cells). (H) There is no GHSR signal in CV of GHSR null mice, illustrating specificity of the GHSR antibody. Scale bars, 20 µm. Blue is TO-PRO-3 nuclear stain.
(2.79 MB TIF)

Figure S3 Co-expression of ghrelin and taste cell markers in circumvallate papillae of GHSR null mice. (A) ghrelin and PLCβ2 are co-localized in a subset of PLCβ2-positive cells. Arrows, cells expressing both; arrowhead, cell expressing ghrelin only. (B) ghrelin and α-gustducin are co-localized in a subset of α-gustducin-positive cells. Arrows, cells expressing both; arrowhead, cell expressing ghrelin only. (C) ghrelin and NCAM are co-localized in a subset of NCAM-positive cells. Arrows, cells expressing both. (D) ghrelin and PGP9.5 are co-localized in a subset of PGP9.5-positive cells. Arrows, cells expression both; arrowhead, cell expressing ghrelin only. (E) ghrelin and Shh are co-localized in a subset of Shh-positive cells. Arrow, cell expressing expressing both; arrowhead, cell expressing ghrelin only. Scale bars, 20 µm. Blue is TO-PRO-3 nuclear stain.
(5.56 MB TIF)

Figure S4 Altered salt and sour taste responses of WT and GHSR null mice in brief access taste tests (A–D). Taste responses, expressed as tastant/water lick ratios and as a function of stimulus concentration, of GHSR null, (red) and WT (black) to (A) sucrose, (B) denatonium benzoate (DB), (C) NaCl and (D) citric acid (CA). Points are expressed as means ± S.E.M. Curves were fitted as described in Methods. Mean calculated log EC50 values (± S.E.M) for WT or GHSR knockout are depicted in associated histograms with each panel. The specific log EC50 values are as follows: (A) sucrose, WT = −0.7602 ± 0.028, KO = −0.7553±0.023; (B) DB, WT = −1.69±0.0718, KO = −1.671±0.08; (C) NaCl, WT = −0.573±0.0285, KO = −0.4639±0.0227; (D) CA, WT = −2.66±0.0534, KO = −2.229±0.0541. **p<0.01; ***p<0.001.
(1.12 MB TIF)

Figure S5 Co-expression of ghrelin and GHSR with NTPDase2 in mouse fungiform papillae (A,B) and ghrelin and GHSR immunostaining in monkey CV taste cells (C, D). (A) Ghrelin is co-expressed with NTPDase2. Arrows, cells expressing both. (B) GHSR is co-expressed with NTPDase2. Arrows, cells expressing both. (C, D) monkey CV. Scale bars, 20 µm. Blue is TO-PRO-3 nuclear stain.
(3.56 MB TIF)

Table S1 Sequences and efficiency of primers employed for RT-PCR amplifications.
(0.06 MB DOC)

Table S2 Primary antibodies used in immunofluorescence analyses.
(0.06 MB DOC)

Table S3 Co-localization of immunocytochemical markers in mouse foliate papillae taste cells.
(0.03 MB DOC)

Table S4 Immunocytochemical markers in mouse foliate papillae taste cells.
(0.03 MB DOC)
