# Supplementary figures and images for: Ghrelin Is Produced in Taste Cells and Ghrelin Receptor Null Mice Show Reduced Taste Responsivity to Salty (NaCl) and Sour (Citric Acid) Tastants
Source: PLoS One. 2010 Sep 14;5(9):e12729. doi: 10.1371/journal.pone.0012729 (PMC2939079; doi:10.1371/journal.pone.0012729)

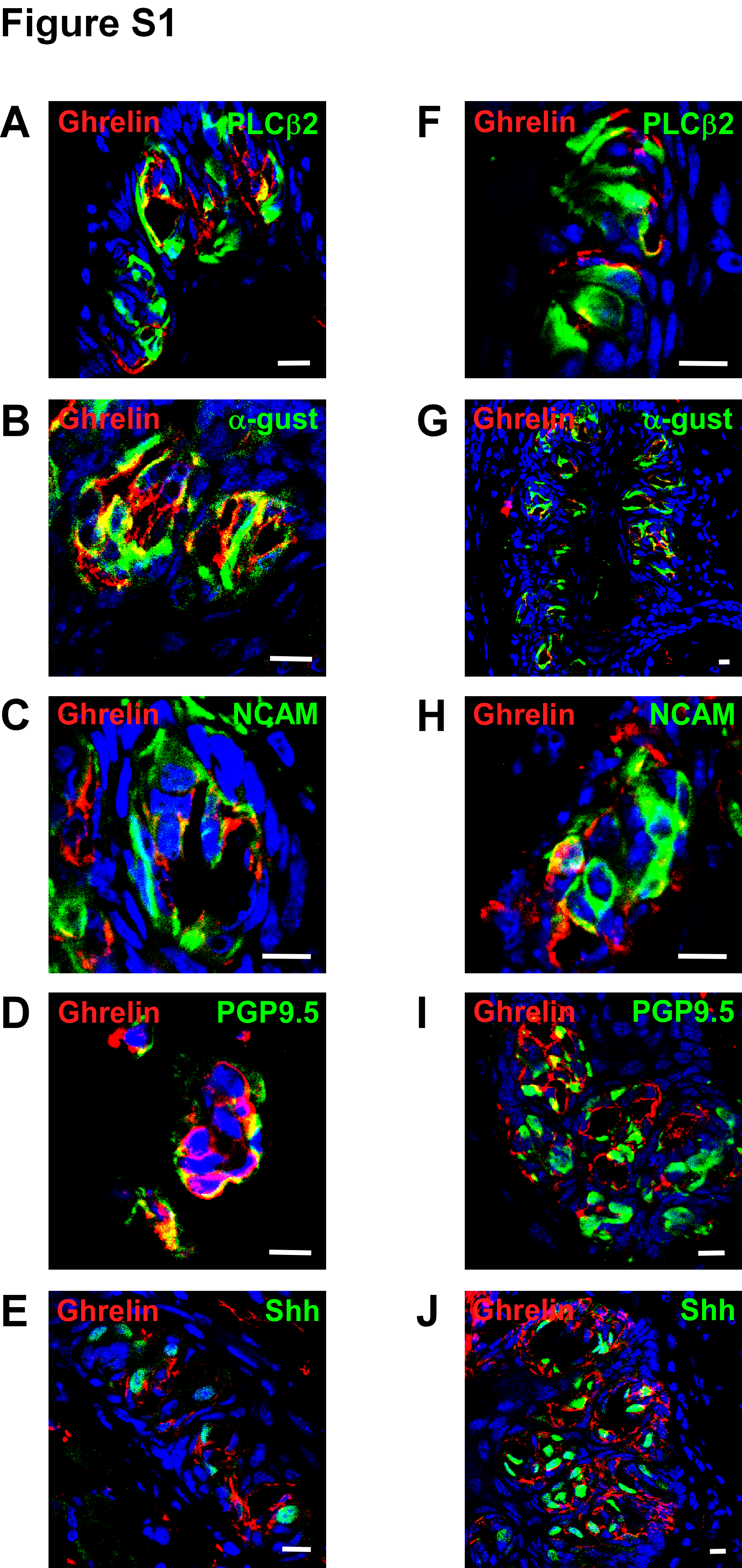

Supplement: Figure S1 — Co-expression of ghrelin in mouse foliate papillae of WT (A–E) and GHSR null (F–J) mice. (A, F) ghrelin is co-expressed with PLCβ2. (B, G) ghrelin is co-expressed with α-gustducin. (C, H) ghrelin is co-expressed with NCAM. (D, I) ghrelin is co-expressed with PGP9.5. (E, J) ghrelin is co-expressed with Shh. Scale bars, 20 µm. Blue is TO-PRO-3 nuclear stain. (3.68 MB TIF) [file pone.0012729.s001.tif]

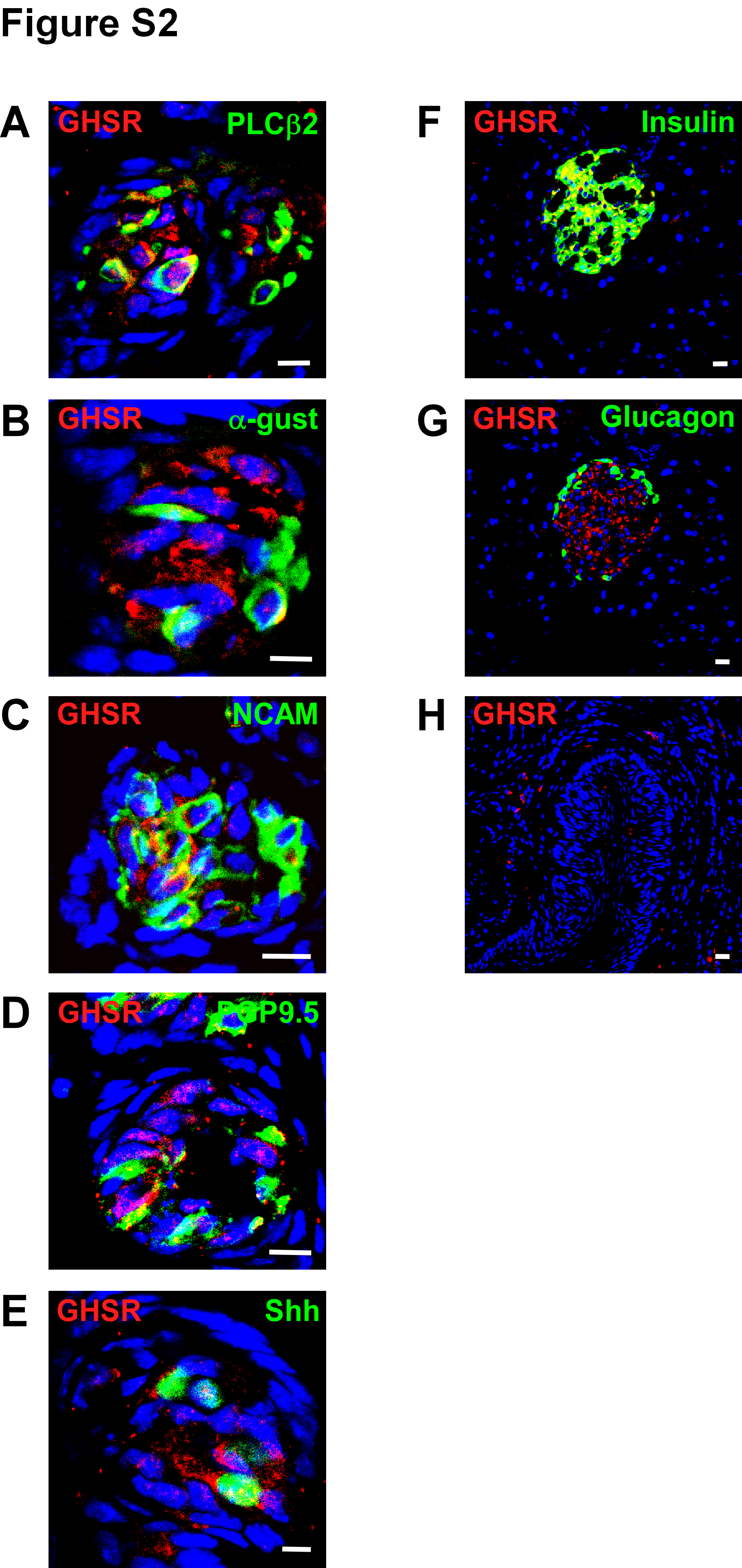

Supplement: Figure S2 — Co-expression of GHSR in foliate papillae of wild type (A–E) and islets of Langerhans (F–H) of wild type and GHSR null mice. (A) GHSR is co-expressed with PLCβ2. (B) GHSR is co-expressed with α-gustducin. (C) GHSR is co-expressed with NCAM. (D) GHSR is co-expressed with PGP9.5. (E) GHSR is co-expressed with Shh. (F) In islets, GHSR is co-expressed with insulin-containing cells (yellow); therefore in islets GHSR is expressed on cells. (G) GHSR (red) is not expressed in glucagon-containing (green) cells (no yellow cells). (H) There is no GHSR signal in CV of GHSR null mice, illustrating specificity of the GHSR antibody. Scale bars, 20 µm. Blue is TO-PRO-3 nuclear stain. (2.79 MB TIF) [file pone.0012729.s002.tif]

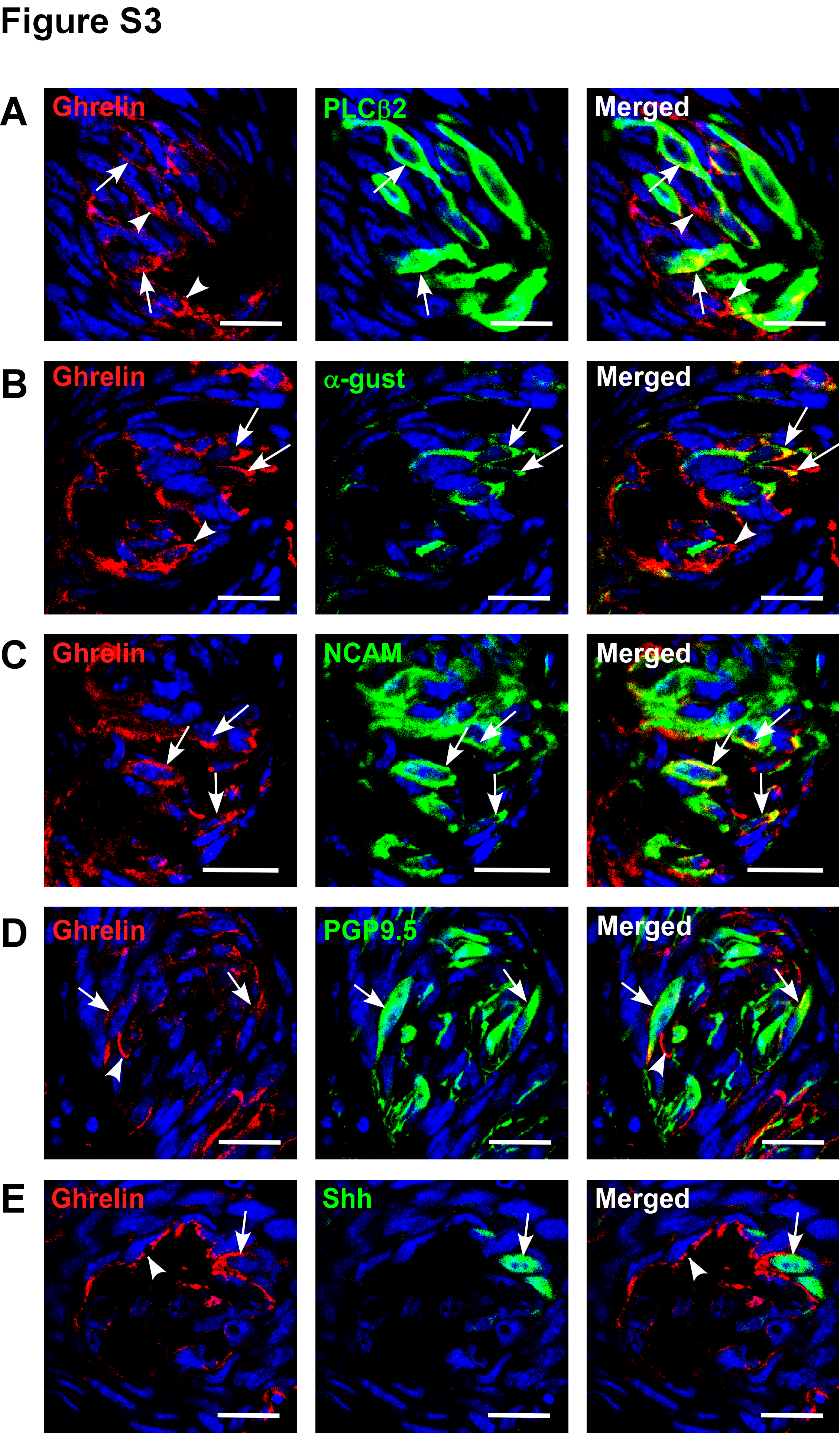

Supplement: Figure S3 — Co-expression of ghrelin and taste cell markers in circumvallate papillae of GHSR null mice. (A) ghrelin and PLCβ2 are co-localized in a subset of PLCβ2-positive cells. Arrows, cells expressing both; arrowhead, cell expressing ghrelin only. (B) ghrelin and α-gustducin are co-localized in a subset of α-gustducin-positive cells. Arrows, cells expressing both; arrowhead, cell expressing ghrelin only. (C) ghrelin and NCAM are co-localized in a subset of NCAM-positive cells. Arrows, cells expressing both. (D) ghrelin and PGP9.5 are co-localized in a subset of PGP9.5-positive cells. Arrows, cells expression both; arrowhead, cell expressing ghrelin only. (E) ghrelin and Shh are co-localized in a subset of Shh-positive cells. Arrow, cell expressing expressing both; arrowhead, cell expressing ghrelin only. Scale bars, 20 µm. Blue is TO-PRO-3 nuclear stain. (5.56 MB TIF) [file pone.0012729.s003.tif]

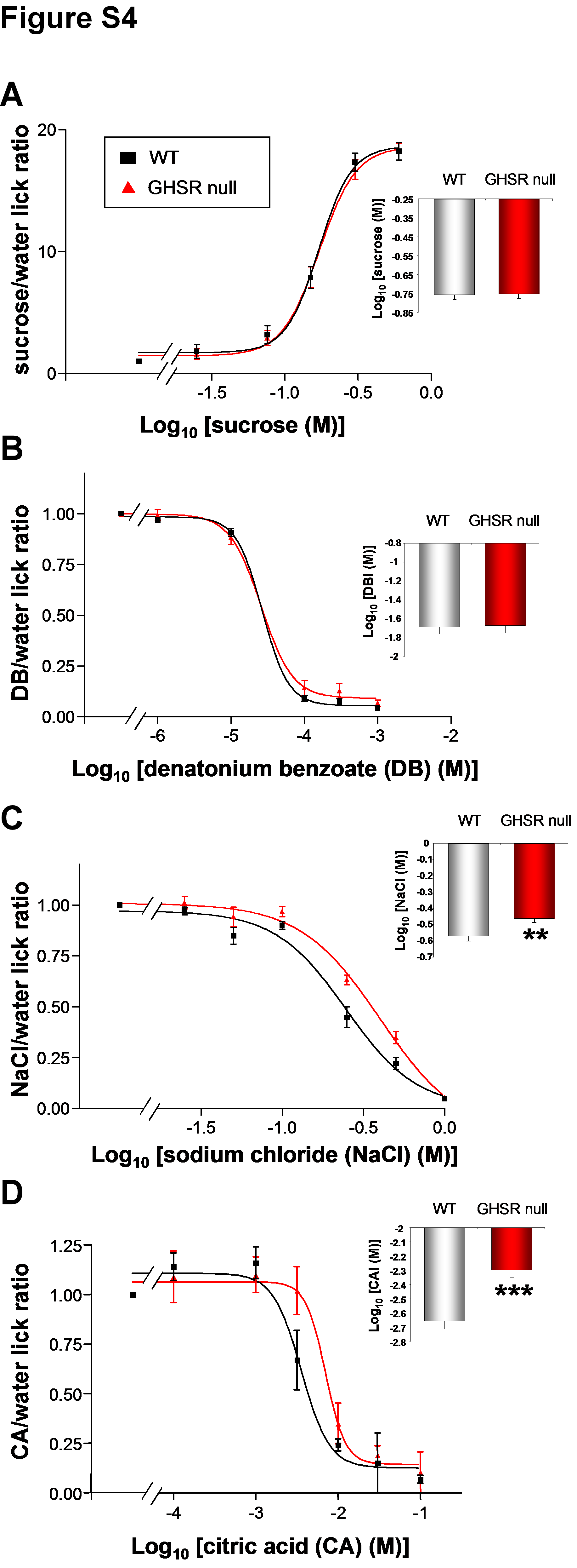

Supplement: Figure S4 — Altered salt and sour taste responses of WT and GHSR null mice in brief access taste tests (A–D). Taste responses, expressed as tastant/water lick ratios and as a function of stimulus concentration, of GHSR null, (red) and WT (black) to (A) sucrose, (B) denatonium benzoate (DB), (C) NaCl and (D) citric acid (CA). Points are expressed as means ± S.E.M. Curves were fitted as described in Methods. Mean calculated log EC50 values (± S.E.M) for WT or GHSR knockout are depicted in associated histograms with each panel. The specific log EC50 values are as follows: (A) sucrose, WT = −0.7602 ± 0.028, KO = −0.7553±0.023; (B) DB, WT = −1.69±0.0718, KO = −1.671±0.08; (C) NaCl, WT = −0.573±0.0285, KO = −0.4639±0.0227; (D) CA, WT = −2.66±0.0534, KO = −2.229±0.0541. **p<0.01; ***p<0.001. (1.12 MB TIF) [file pone.0012729.s004.tif]

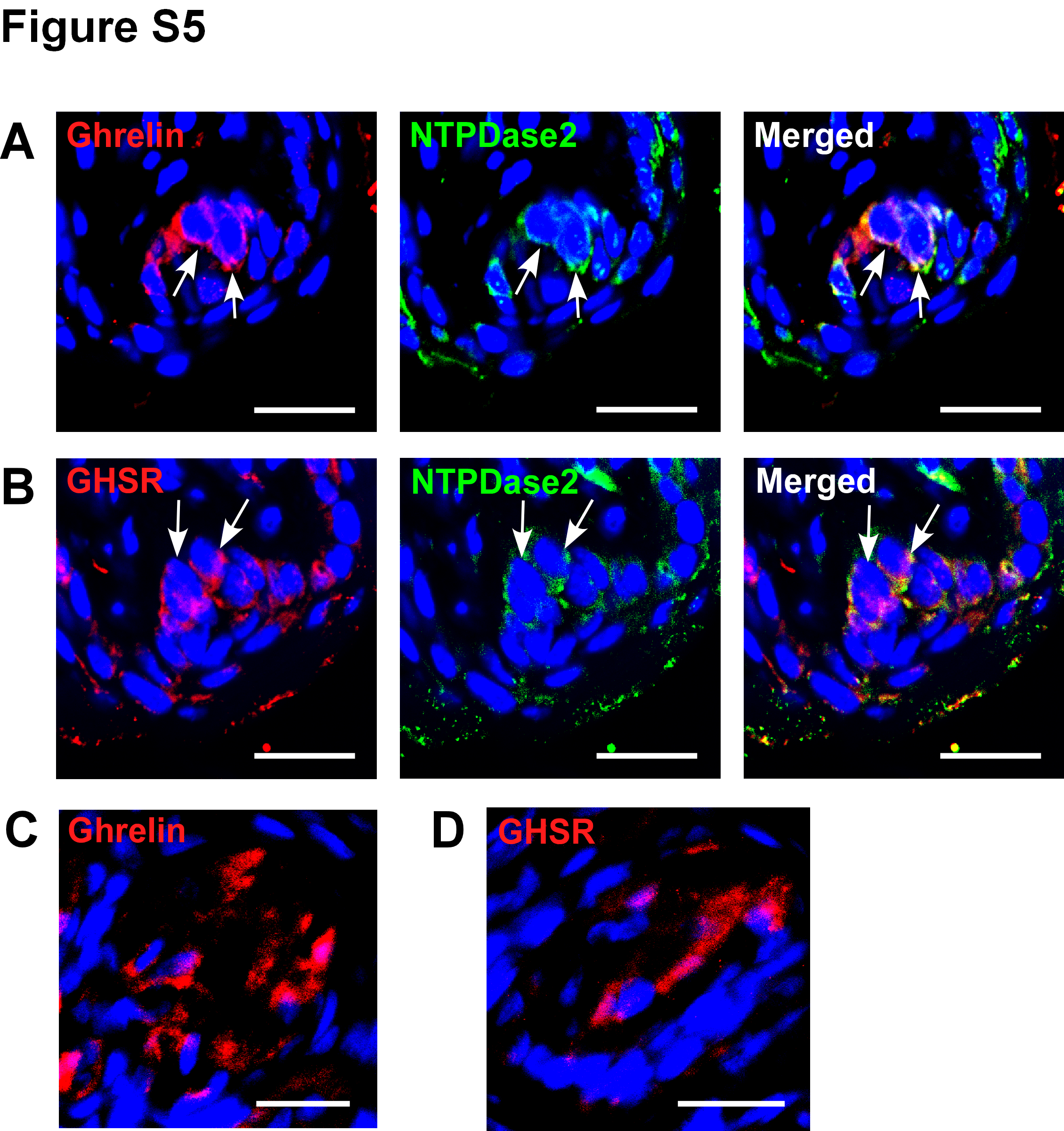

Supplement: Figure S5 — Co-expression of ghrelin and GHSR with NTPDase2 in mouse fungiform papillae (A,B) and ghrelin and GHSR immunostaining in monkey CV taste cells (C, D). (A) Ghrelin is co-expressed with NTPDase2. Arrows, cells expressing both. (B) GHSR is co-expressed with NTPDase2. Arrows, cells expressing both. (C, D) monkey CV. Scale bars, 20 µm. Blue is TO-PRO-3 nuclear stain. (3.56 MB TIF) [file pone.0012729.s005.tif]
