# Supplementary material for: Ghrelin Is Produced in Taste Cells and Ghrelin Receptor Null Mice Show Reduced Taste Responsivity to Salty (NaCl) and Sour (Citric Acid) Tastants
Source: PLoS One. 2010 Sep 14;5(9):e12729. doi: 10.1371/journal.pone.0012729 (PMC2939079; doi:10.1371/journal.pone.0012729)
Supplement: Table S1 — Sequences and efficiency of primers employed for RT-PCR amplifications. (0.06 MB DOC) [file pone.0012729.s006.doc]

**Table S1. Sequences and efficiency** of primers employed for RT-PCR amplifications.

| **Measurement** | **Forward Primer** | **Reverse Primer** |
| --- | --- | --- |
| T1R2 (AY032623.1) | ATGGCAGCTACTCAGGGAGA | GAGTAGGAGGCGATGCTTTG |
| Ghrelin (NM_021488) | CCATCTGCAGTTTGCTGCTA | GCTTGTCCTCTGTCCTCTGG |
| GOAT (XM_001476434) | GGGCCAGGTACCTCTTTCTC | GCCTATGGACTTCCTGTGGA |
| GHSR 1a (AY255570) | TCCGATCTGCTCATCTTCCT | GGAAGCAGATGGCGAAGTAG |
| GAPDH (BC145810) | AACTTTGGCATTGTGGAAGG | GGATGCAGGGATGATGTTCT |
