# Supplementary material for: Ghrelin Is Produced in Taste Cells and Ghrelin Receptor Null Mice Show Reduced Taste Responsivity to Salty (NaCl) and Sour (Citric Acid) Tastants
Source: PLoS One. 2010 Sep 14;5(9):e12729. doi: 10.1371/journal.pone.0012729 (PMC2939079; doi:10.1371/journal.pone.0012729)
Supplement: Table S2 — Primary antibodies used in immunofluorescence analyses. (0.06 MB DOC) [file pone.0012729.s007.doc]

**Table S2. Primary antibodies used in immunofluorescence analyses.**

| **Antigen** | **Host** | **Vendor** | **Dilution** | **Ref.** |
| --- | --- | --- | --- | --- |
| Preproghrelin | Rabbit | Phoenix, Belmont, CA | 1:100 | 35 |
| Ghrelin | Goat | Santa-Cruz, Santa Cruz, CA | 1:100 | 36 |
| Ghrelin | Rabbit | Phoenix, Burlingame, CA | 1:200 | 35 |
| Growth Hormone Secretagogue Receptor 1A (GHS-R 1A) | Goat | Santa-Cruz, Santa Cruz, CA | 1:100 | 35 |
| Growth Hormone Secretagogue Receptor 1A (GHS-R 1A) | Rabbit | Phoenix, Burlingame, CA | 1:200 | 37 |
| Ghrelin O-Acyltransferase (GOAT) | Rabbit | Phoenix, Burlingame, CA | 1:100 | 38 |
| NTPDase2 | Rabbit | Provided by Dr. Sévigny University of Laval, QC | 1:100 | 39 |
| Phospholipase C_β_2 (PLCβ2) | Rabbit | Santa-Cruz, Santa Cruz, CA | 1:200 | 40,41 |
| α-gustducin | Rabbit | Santa-Cruz, Santa Cruz, CA | 1:200 | 40,41 |
| Neural Cell Adhesion Molecule (NCAM) | Rabbit | Chemicon, Temecula, CA | 1:500 | 42 |
| Protein Gene Product 9.5 (PGP9.5) | Rabbit | Biogenesis, Raleigh, NC | 1:200 | 40 |
| Sonic Hedgehog (Shh) | Rabbit | Santa-Cruz, Santa Cruz, CA | 1:100 | 8 |
| Prohormone convertase 1/3 (PC 1/3) | Rabbit | Provided by Dr DF Steiner, University of Chicago, IL | 1:200 | 25 |
| Anti-Epithelial Sodium Channel-alpha (ENaCα) | Rabbit | Chemicon, Temecula, CA | 1:100 | 43 |
| Anti-Epithelial Sodium Channel-gamma (ENaCγ) | Rabbit | Chemicon, Temecula, CA | 1:100 | 43 |

**Type II**

**Type III**

**Type IV**


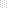


**PLCβ2**

**IP3R3**

**TrpM5**

**α-gustducin**

**(subset)**

**PGP9.5 (subset)**

**T1Rs**

**T2Rs**

**Proglucagon**

**NCAM**

**SNAP-25**

**5-HT (subset)**

**PGP9.5 (subset)**

**PKD2L1**

**PLCβ2 (subset)**

**Proglucagon**

**Sonic Hedgehog**


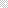


**NTPDase2**

**GLAST**

**CK-7**

**Ck-19**

**Type I**
