# Supplementary material for: Ghrelin Is Produced in Taste Cells and Ghrelin Receptor Null Mice Show Reduced Taste Responsivity to Salty (NaCl) and Sour (Citric Acid) Tastants
Source: PLoS One. 2010 Sep 14;5(9):e12729. doi: 10.1371/journal.pone.0012729 (PMC2939079; doi:10.1371/journal.pone.0012729)
Supplement: Table S3 — Co-localization of immunocytochemical markers in mouse foliate papillae taste cells. (0.03 MB DOC) [file pone.0012729.s008.doc]

**Table S3. Co-localization of immunocytochemical markers in mouse foliate papillae taste cells.**

| Co-localized Marker | Co-localized taste cells / total ghrelin-positive taste cells | |
| --- | --- | --- |
|  | (Percentages, Mean ± S.E.M.) | |
|  | WT mice | GHSR null mice |
| PLCβ2 | 28 ± 6 | 28 ± 3 |
| α-gustducin | 26 ± 8 | 28 ± 8 |
| NCAM | 36 ± 9 | 40 ± 4 |
| PGP9.5 | 18 ± 6 | 16 ± 4 |
| Shh | 13 ± 2 | 13 ± 2 |
| Rest of TCs | 23 ± 2 | 19 ± 2 |
