# Supplementary material for: Ghrelin Is Produced in Taste Cells and Ghrelin Receptor Null Mice Show Reduced Taste Responsivity to Salty (NaCl) and Sour (Citric Acid) Tastants
Source: PLoS One. 2010 Sep 14;5(9):e12729. doi: 10.1371/journal.pone.0012729 (PMC2939079; doi:10.1371/journal.pone.0012729)
Supplement: Table S4 — Immunocytochemical markers in mouse foliate papillae taste cells. (0.03 MB DOC) [file pone.0012729.s009.doc]

**Table S4. Immunocytochemical markers in mouse foliate papillae taste cells.**

| Marker | Cell type | Immunoreactive taste cells / total taste cells | |
| --- | --- | --- | --- |
|  |  | (Percentages, Mean ± S.E.M.) | |
|  |  | WT mice | GHSR null mice |
| PLCβ2 | II | 23 ± 1 | 25 ± 4 |
| α-gustducin | II | 18 ± 1 | 20 ± 1 |
| NCAM | III | 31 ± 2 | 34 ± 2 |
| PGP9.5 | II & III | 9 ± 1 | 10 ± 1 |
| Shh | IV | 3 ± 0.1 | 3 ± 0.2 |
| Ghrelin |  | 13 ± 2 | 13 ± 2 |
